# Exploring arbuscular mycorrhizal colonization in *Agave tequilana*: Insights into mycorrhizal partnerships of an emerging crop

**DOI:** 10.1007/s00572-025-01225-4

**Published:** 2025-08-27

**Authors:** Matthias Johannes Salomon, Rachel Anita Burton

**Affiliations:** The School of Agriculture, Food and Wine, The University of Adelaide Waite Campus, Adelaide, PMB1 Glen Osmond, SA 5064 Australia

**Keywords:** *Agave Tequilana*, *Plantago lanceolata*, Metagenomic sequencing, Sustainable agriculture, AMF, Root colonization

## Abstract

This study explores the arbuscular mycorrhizal (AM) fungi associations of *Agave tequilana*, an emerging crop with significant commercial potential that is increasingly being grown outside its native distribution in the arid regions of the Americas. A greenhouse experiment was conducted using field-collected soil inoculum from various locations in South Australia to inoculate *A. tequilana* plus *Plantago lanceolata* as a comparative model host. Metagenomic sequencing of the colonized roots with AM fungal-specific amplicons assessed the AM fungal community structure. The Shannon alpha diversity was similar between both plant species, and both predominantly associated with the genus *Glomus*. However, *A. tequilana* had a narrower AM fungal community compared to *P. lanceolata*, which was associated with a broader diversity of AM fungi. Beta diversity analysis of the AM fungal community composition revealed that the factor species (agave/plantago) was significant (*p* = 0.0001), whereas the inoculum location was not (*p* = 0.29). The association of *A. tequilana* with AM fungi from the family Acaulosporaceae, as found in Mexican studies, could not be confirmed. These findings suggest a specialized adaptation of *A. tequilana* to specific AM fungal species, which could have important implications for using AM fungi in sustainable agave production when cultivated outside their native regions.

**Supplementary Information:**

The online version contains supplementary material available at 10.1007/s00572-025-01225-4.

## Highlights

*Agave tequilana* associates mainly with the *Glomus* genus.

Agave shows narrower AM fungal beta diversity compared to *Plantago lanceolata*.

AM fungal community structure in Australian-grown agave diverges from patterns observed in Mexican studies.

Need for research to support agave cultivation with AM fungi in non-native areas.

**Supplementary Information:**

The online version contains supplementary material available at 10.1007/s00572-025-01225-4.

## Introduction

*Agave* is a genus of plants within the family Asparagaceae, comprising about 270 documented species. They are native to the Americas and primarily found in (semi-)arid and temperate regions (García-Mendoza 2011). Agave has numerous traditional uses, but the predominant commercial use is for spirit production, notably tequila, which is made from *Agave tequilana* (Pérez-Zavala et al. 2020). Agave plants are highly resilient in adverse environmental conditions, being resistant to many biotic and abiotic stress factors. They can thrive on marginal land with limited soil nutrients and rainfall and cope well with the effects of climate change, such as elevated temperatures and CO_2_ (Davis et al. 2021). Under good growing conditions, many agave species are extremely productive (Nobel et al. 1992), with total aboveground biomass comparable to other high yielding crops such as sugarcane or corn (Yan et al. 2011; Núñez et al. 2011). Given the increase in extreme weather events due to climate change and the overarching need to reduce agriculture’s environmental footprint, agave has been proposed as a novel crop with substantial commercial potential (Crawford et al. 2022).

Arbuscular mycorrhizal (AM) fungi are a group of symbiotic fungi that form mutualistic associations with the roots of most terrestrial plants (Oliveira et al. 2024). These fungi play a crucial role in natural ecosystems and have been described as a driving force behind soil health, as well as above and belowground biodiversity (Wang and Rengel 2024). In agricultural ecosystems, AM fungi can contribute to increased crop yields and enhanced quality by increasing the plant’s nutrient uptake, improving drought resistance, soil health and overall plant productivity whilst reducing disease pressure (Wang and Rengel 2024). In the context of agave, which is an emerging and introduced crop in many countries outside its native area of the Americas, understanding its relationship with AM fungi presents a beneficial pathway for increased production (Rouphael et al. 2015). Since agave are mostly propagated in nurseries before being transplanted into the field, this presents a good opportunity to deliberately inoculate plants with specific AM fungal species to enhance their growth (Yan et al. 2011; Tawaraya et al. 2012). However, before tailoring the mycorrhizal community used as agave inoculum, it is essential to first understand the specific AM fungal relationships that agave forms.

There is existing knowledge about agave and its AM fungal preferences, but all previous studies have been conducted in Mexico. These studies have focused on a range of agave species, rather than specifically on the economically significant *A. tequilana*. Generally, *Agave sp.* roots are strongly colonized by AM fungi, at up to 83% of root length (Carballar-Hernández et al. 2013). Several studies have investigated agave’s AM fungal community composition and molecular methods have identified Ambispora and Glomus as the dominant genera (Chávez‐González et al. 2024). Other studies also reported Glomus as the prevailing genus but accompanied by a notable abundance of Acaulosporceae (up to 35%) and Gigasporaceae (up to 27%) (Celerino Robles et al. 2010; Carballar‐Hernández et al. 2013; Trinidad-Cruz et al. 2017; Reyes-Jaramillo et al. 2019). The relative abundance levels between AM genera were found to vary depending on habitat, sampling time, and host species (Celerino Robles et al. 2010; Trinidad-Cruz et al. 2017).

While agave’s AM fungal communities have been well studied in Mexico, there is a notable gap for AM fungal associations of plants grown outside their native range, especially for the commercially important species *A. tequilana*. Even though most AM fungal species are cosmopolitan, the distribution of AM families differs markedly across climatic zones and continents (Davison et al. 2015; Stürmer et al. 2018). Several Mexican studies showed the positive impact of AM fungi on agave cultivation, highlighting increased drought resistance, growth and nutrient uptake (Montoya-Martínez et al. 2019; Hernández-Cuevas et al. 2023; Chávez-González et al. 2024). So, it is evident that AM fungi play a critical role in crop success. It is also clear that AM fungal community composition and function can vary across continents; thus, agave’s AM symbiosis is unexplored in the broader geographical context.

This study is the first to investigate the mycorrhizal symbiosis of *A. tequilana* outside its native range in Mexico. The primary objective is to identify AM fungal community composition and host preferences, rather than assessing functional outcomes (e.g., plant growth or drought tolerance). We hypothesized that despite geographic differences, AM fungal colonization patterns would resemble those observed in Mexican studies, with members of the genus *Glomus* predominating in the roots of *A. tequilana*. To test this, a controlled greenhouse experiment was conducted with field soil inoculum collected from multiple Australian locations. Each inoculum was applied to *Plantago lanceolata* as a reference host plant, chosen due to its cosmopolitan distribution, association with a broad spectrum of AM fungal taxa and common use as an AM fungal trap plant (Oehl et al. 2003; Fontana et al. 2009; Velázquez and Cabello 2011; Wang et al. 2015). AM fungal colonization was assessed using quantitative root staining and advanced amplicon-based metagenomic sequencing, using AMF-specific amplicons targeting the small subunit ribosomal RNA gene region.

## Materials and methods

### Field soil inoculum

**Fig. 1:**
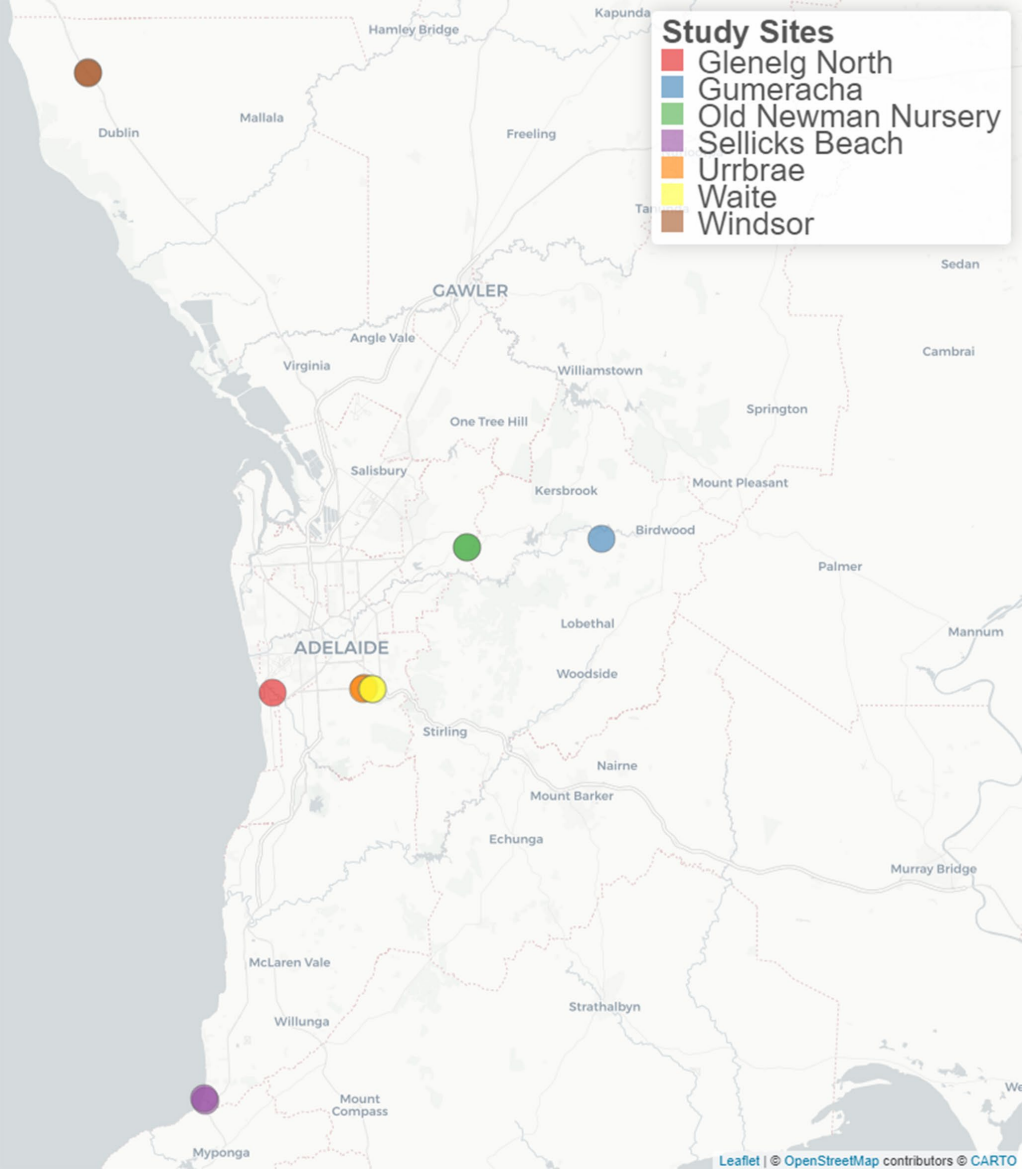
Map highlighting sampling locations of collected field soil inoculum from naturalised Agave patches in proximity to Adelaide, South Australia. The basemap is provided by openstreetmap contributors and is visualized using Leaflet. © openstreetmap contributors

In July 2022, field soil was collected from naturalized agave patches around Adelaide, South Australia. Several locations were chosen, containing predominantly *A. americana*, with smaller populations of *A. angustifolia* and *A. attenuata* (Fig. 1; Table 1). These agave plants, initially cultivated in Australia as ornamental species, have persisted for decades and subsequently naturalized in local patches. Although considered invasive and resilient plants, there is no widespread distribution in South Australian ecosystems due to the lack of suitable pollinators (Holtum et al. 2011; State Herbarium of South Australia 2024). These sites were not selected because they represent future agave cultivation zones, but rather because they harbor long-established agave populations likely to have coexisted with AM fungal communities naturally occurring in South Australian soils. The *A. tequilana* patch at the Waite research site was the only managed planting, established in 2021 for experimental purposes and not representative of a naturalized population.

**Table 1:** Table provides details on the field soil inoculum used for the greenhouse experiment, including its collection location, associated Agave species, dominant cover crop, and results from soil chemical analysis conducted immediately after inoculum collection. The percentage of root colonization reflects outcomes from the greenhouse experiment using micropropagated plants after 243 days growth period. Colwell P = plant-available phosphorus, EC = electrical conductivity

Sample	Location	Agave species	Cover crop	pH	EC (µS cm^−1^)	NH_4_-N (mg kg^−1^)	NO_3_-N (mg kg^−1^)	Colwell P (mg kg^−1^)	Agave root colonization (%)	Plantago root colonization (%)
1	Glenelg North	*A. attenuate*	*Lolium perenne* L. (rye grass)	7.8	96.3	0.0	2.2	18.7	99%	58%
2	Old Newman Nursery	*A. americana*	*Oxalis pes-caprae* L. (sourgrass)	7.2	111.4	3.8	6.6	35.7	78%	4%
3	Old Newman Nursery 2	*A. americana*	*Oxalis pes-caprae* L. (sourgrass)	7.1	116.0	1.3	10.4	52.4	61%	3%
4	Windsor	*A. americana*	*Rytidosperma* sp. (wallaby grass)	7.8	114.0	0.6	0.0	12.6	88%	9%
5	Windsor	*A. angustifolia*	*Rytidosperma* sp. (wallaby grass)	7.7	45.2	1.7	0.1	12.4	43%	26%
6	Gumeracha	*A. americana*	*Rytidosperma* sp. (wallaby grass)	6.5	61.0	3.6	7.9	8.9	77%	61%
7	Sellicks Beach	*A. americana*	*Oxalis pes-caprae* L. (sourgrass)	6.7	109.8	6.2	7.5	26.6	36%	72%
8	Sellicks Beach	*A. americana*	*Oxalis pes-caprae* L. (sourgrass)	6.7	404.0	12.8	0.7	24.6	81%	39%
9	Urrbrae	*A. angustifolia*	*Oxalis pes-caprae* L. (sourgrass)	7.5	125.2	1.1	3.8	19.0	77%	56%
10	Urrbrae	*A. angustifolia*	*Oxalis pes-caprae* L. (sourgrass)	7.2	151.7	0.9	4.9	0.0	91%	1%
11	Waite	*A. tequilana*	*Microlaena* sp. (weeping grass)	6.9	75.7	1.1	2.9	0.0	83%	40%
12	Waite	*A. tequilana*	*Microlaena* sp. (weeping grass)	6.5	73.4	0.2	1.8	0.0	65%	40%

The soil was collected from the 0–10 cm layer near the plant base. Five soil samples were collected and homogenized and depending on the size of the agave patch, one or two aliquots were dug up from opposite ends of the site. In all locations, agave plants grew amongst ground cover plants, indicating that the AM fungal community in the collected soil relates to a mix of agave and at least one other plant type (Table 1). About 500 g of dried soil equivalent was collected from each location and kept cool until further analysis. In the lab, soils were subsampled and analyzed as follows. The first subsample was taken to measure moisture content of the fresh soil by drying it at 105 °C for at least 24 h. The second subsample was used to determine the concentration of mineral nitrogen (ammonium and nitrate). Five grams of fresh soil were collected in duplicates and extracted in 2 M potassium chloride (KCl) by shaking at 125 rpm for 60 min. The extractant was analyzed colorimetrically (Salomon et al. 2025). The remaining soil was dried at 40 °C for at least 48 h, then sieved to 2 mm and used to determine pH and electric conductivity (EC) using a calibrated TPS WP-81 pH/EC/temperature meter (TPS Pty Ltd., Brisbane, QLD, Australia) (Rayment and Lyons 2010). Additionally, plant-available (Colwell) phosphorus (P) was measured using a 1:100 ratio of soil to extractant made from 0.5 M sodium bicarbonate at pH 8.5. The extractant was then neutralized using 0.5 M hydrochloric acid (HCl) and concentration of P was determined colorimetrically (Murphy and Riley 1986). The remaining soil was air-dried and stored at room temperature for three weeks before being used as the inoculum in the greenhouse experiment.

### Greenhouse setup

The selected plant substrate was low in P and has been shown in a previous study to be favorable for AM fungal colonization (Salomon et al. 2022). It was made from a mix of sand and soil in a 70:30 ratio. The soil was collected from the Waite Arboretum (−34.968596, 138.631956) in September 2022 from the 0–10 cm soil layer and is classified as Urrbrae red-brown earth (Alfisol). It was air-dried and sieved to 2 mm. After mixing with sand, it was sterilized by double autoclaving at 121 °C for 60 min, with one day between the first and second autoclaving. Pots were clean uncontaminated 1.5-litre ice cream buckets with three air holes drilled into the base. These pots have thicker plastic walls than standard plant pots, which are more suitable for longer greenhouse experiments. Each pot was filled with 1,675 g of sterilized substrate and 125 g of air-dried field inoculum. According to the experimental design, each field inoculum sample was split in two, with one half used for growing *A. tequilana* and the other half for growing *P. lanceolata* as the comparison model plant. The substrate was overall low in plant nutrients and chemical analysis of the soil before mixing with sand was pH = 4.63, EC = 80.1 µS cm^−1^, NH_4_^+^ = 2.1 mg kg^−1^, NO_3_^−^ = 1.6 mg kg^−1^, plant-available (Colwell) *P* = 4.6 mg kg^−1^, total C = 1.81%, total *N* = 0.16%.

Micropropagated plantlets (clonal) of *A. tequilana* were provided by Chris Monsour (Prospect Agriculture, Queensland, Australia) and Topshelf International and are genetically identical to the plants grown at the commercial Eden Lassie Agave Farm in Bowen, Queensland. Plants arrived in September 2022 in 500 mL plastic containers, when they were deflasked and their roots washed free of agar. Roots were subsequently trimmed to a maximum length of 2 cm and planted into the pots. *P. lanceolata* was grown from seeds with five seeds added to each pot. Seeds were surface sterilized by stirring in 2% sodium hypochlorite (NaClO) for 5 min and then thoroughly rinsed in water. Each pot was placed inside a Sun Bag (Sigma-Aldrich, B7026; Merck KGaA, Darmstadt, Germany) with a 0.02 μm filter patch. This setup reduced the chances of contamination with foreign AM fungi and allowed for sufficient air exchange for plant growth (Walker and Vestberg 1994). Subsequently, the seedlings were moved to the greenhouse with an average daily temperature of 22 °C and allowed to grow for 243 days. Every second week, 10 mL of modified Long Ashton nutrient solution without phosphorus (P) was added and pots watered to 10% gravimetric moisture content using reverse osmosis (RO) water, allowing good plant growth under conditions favorable to AM fungal colonization (Salomon et al. 2022).

### Experimental design

Each inoculum sample (*n* = 12) was applied to a single replicate pot for *A. tequilana* and one for *P. lanceolata*, resulting in 24 experimental units. They were placed in the greenhouse as a complete randomized design and randomized weekly. Each inoculum was represented by only one replicate per plant species, reflecting the deliberate choice to prioritize geographical breadth over replication (i.e. maximizing the number of independent samples rather than depth per sample). This design limits statistical inference due to the lack of replication but increases AM fungal diversity across the experiment. This design was selected to capture the widest possible range of naturally assembled AM fungal communities across different field sites, rather than to estimate within-site variability (Hermans et al. 2019). Given the exploratory nature of the study and the absence of prior data on AM fungal associations in *A. tequilana* outside its native range, this approach allowed for a broad landscape-scale survey of AM fungal colonization patterns and potential host preferences, within the constraints of high-throughput sequencing costs (see Discussion).

### Harvest

At 243 days after planting, the pots were moved to the laboratory for harvest. This was conducted as follows: first, plants were removed from the Sun Bag and then carefully extracted from the pots by tipping them upside down. Roots were washed free of soil using tap water, rinsed with deionized (DI) water, and then separated from the shoots. For agave plants, root and shoot biomasses were recorded but not discussed further as this study was focused on exploring AM fungal status. Instead, they are only presented as supplementary material (Supplementary Material Table S1). Biomass measurements were not taken for *P. lanceolata* due to the varying number of germinated seeds per pot and significant leaf senescence observed during the growth period. From all pots, a representative root sample of approximately 0.5 g fresh weight was collected from different parts of the root system and stored at −20 °C for subsequent amplicon-based metagenomic sequencing. Another representative subsample of approximately 0.25 g fresh root was collected and stored in 50% ethanol for quantification of root colonization. The remaining root and shoot biomasses were dried at 60 °C for 2 days. Agave shoots were dried for 63 days at 60 °C to ensure complete removal of moisture from the waxy leaves.

Although agave biomass data was measured at harvest (Supplementary Material Table S1), no meaningful statistical analysis on plant growth effects could be performed due to the use of filter bags and experimental design without replications. These bags were used to grow plants without cross-contamination, which does not accurately reflect the realistic conditions of agave cultivation. Furthermore, AM fungal colonization pattern was the focus of this study, rather than biomass data. The study’s ecological focus with increased geographic coverage and no inoculum repetitions limits the ability to draw robust conclusions about plant growth responses.

### AM fungal root colonization quantification

Roots stored in 50% ethanol for staining were first washed with DI water and then cleared in 10% potassium hydroxide (KOH) at room temperature for 4 days (Brundrett et al. 1996). After this period roots were examined under a microscope (Olympus SZ-PT, Tokyo, Japan) at 80x magnification to assess the extent of clearing. As some structures were still visible in the agave roots, they underwent further clearing for 10 min at 70 °C. Following complete clearing, the roots were washed again with DI water and then stained using the ink and vinegar method (Vierheilig et al. 1998) for 15 min at 65 °C. Subsequently, they were destained and stored in acidified water (2% household vinegar diluted with DI water). The percentage colonization by AM fungi was quantified by counting AM structures under the microscope (Olympus SZ-PT, Tokyo, Japan) at 80x magnification using the intersect grid method (McGonigle et al. 1990). Percentage AM fungal root colonization was calculated using the following formula:

$$\begin{array}{c}Root\:colonization\:\left(\%\right)=\\\:\left(\frac{Number\:of\:intersects\:with\:AM\:fungal\:structures}{Total\:number\:of\:intersects\:observed}\right)\times\:100\end{array}\\$$

###  Amplicon sequencing and analysis

 Amplicon sequencing was conducted from root samples stored at −20°C. Samples were submitted to the Australian Genome Research Facility (AGRF, Adelaide, Australia) for DNA extraction, PCR amplification, and Illumina MiSeq sequencing (Illumina Inc., San Diego, CA, USA). In brief, DNA was extracted using the Qiagen DNeasy Plant Pro Kit (Qiagen, Hilden, Germany), following the manufacturer’s instructions. The small-subunit (SSU) ribosomal RNA gene was amplified using the AM fungal-specific primers WANDA-AML2. The primer sequences are 5’-CAGCCGCGGTAATTCCAGCT-3’ (forward, WANDA) and 5’-GAACCCAAACACTTTGGTTTCC-3’ (reverse, AML2) (Frew 2023). After PCR amplification, the amplicons were indexed using a secondary PCR, as described in Smith et al. (2018). The normalized PCR products were then pooled and sequenced on an Illumina MiSeq with 2 × 300 base pairs paired-end reads resulting in at least 50,000 reads per sample. The sequencing data was provided as paired-end FastQ-formatted files and processed as follows.

Primers were removed from FastQ sequences using cutadapt 4.6 implemented in Python 3.12.1 (Martin 2011). Sequences were processed using DADA2 1.30.0 (Callahan et al. 2016) in R 4.3.2 (R Core Team 2024). DADA2’s filterAndTrim function was applied with the following settings to retain enough reads and overlap: maxEE = c(2,2), maxN = 0 and truncQ = 2. Pseudo pooling was employed during the error correction and amplicon sequence variant (ASV) inference step. Taxonomy was assigned using DADA2’s assignTaxonomy function and the 18 S rDNA FASTA release of the MaarjAM database (2021) (Öpik et al. 2010). Before assigning the taxonomy, the MaarjAM database was converted into DADA2’s required fasta format using Python. Statistical analysis to evaluate differences in microbial diversity between *A. tequilana* and *P. lanceolata* was conducted using PERMANOVA with package vegan 2.6-4 (Oksanen et al. 2022).

Rarefaction curves were generated to assess sequencing depth sufficiency and to compare observed ASV richness across samples. ASV tables were processed using the phyloseq (McMurdie and Holmes 2013) and vegan (Oksanen et al. 2022) packages in R. For each sample, the number of observed ASVs was calculated at increasing subsampled sequencing depths, using repeated random subsampling without replacement. Richness was averaged over ten randomizations at each depth to account for stochasticity.

Data was visualized using phyloseq 1.46.0 (McMurdie and Holmes 2013) and ggplot2 3.5.1 (Wickham 2016). The map displaying the sampling locations was created using the packages tmap 3.3-4 (Tennekes 2018) and leaflet 2.2.2 (Cheng et al. 2024) in R, with basemap data sourced from OpenStreetMap and visualized using the CartoDB Positron style.

## Results

### Inoculum soil chemical properties and AM fungal root colonization

The pH of the collected field soil inoculum ranged from 6.5 to 7.8, with EC values between 45.2 and 404.0 µS cm^−1^. Mineral nitrogen and plant-available (Colwell) P were variable but generally low. NH_4_-N ranged from 0 to 12.8 mg kg^−1^ with a mean of 2.8 mg kg^−1^, while NO_3_-N ranged from 0.0 to 10.4 mg kg^−1^ with a mean of 4.1 mg kg^−1^. Plant-available (Colwell) ranged from 0.0 to 52.4 mg kg^−1^ with a mean of 17.6 mg kg^−1^ (Table 1). *A. tequilana* roots harvested after 243 days were consistently highly colonized, ranging from 36 to 99% total root length (mean 73%). In contrast, *P. lanceolata* colonization was more variable, with some samples between 1% and 9% and other samples up to 72% (mean 34%) (Table 1, Supplementary Material Figure S1).

### Alpha and beta diversity

**Fig. 2:**
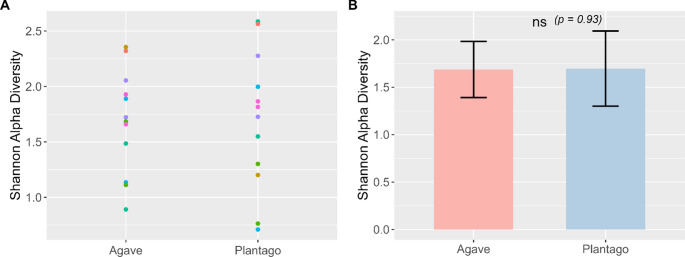
**A** Shannon alpha diversity values for *Agave tequilana* and *Plantago lanceolata* with a single point and different colour representing individual samples. **B** Mean shannon alpha diversity for each plant species. Error bars represent standard error of the mean; “ns” denotes no significant difference between groups (Wilcoxon test)

The Shannon alpha diversity for *A. tequilana* and *P. lanceolata* were within a similar range, from approximately 1.0 to 2.5 (Fig. 2A). The mean Shannon alpha diversity between species and pooled by sample location was 1.69 for *A. tequilana* and 1.70 for *P. lanceolata*. Statistical analysis indicated no significant difference between the two groups (*p* = 0.93) (Fig. 2B). Rarefaction showed that all samples reached or approached a plateau (Supplementary Material Figure F2). However, there was considerable variation in ASV richness between samples, with the maximum observed ASVs in any single sample ranging from 65 to over 220. The mean maximum richness between *A. tequilana* and *P. lanceolata* was very similar (167 and 170), reflecting the results from the Shannon alpha diversity. The maximum sequencing depth for *A. tequilana* samples was 250,076 reads with 216,557 reads for *P. lanceolata* samples (Supplementary Material Table S2).

**Fig. 3:**
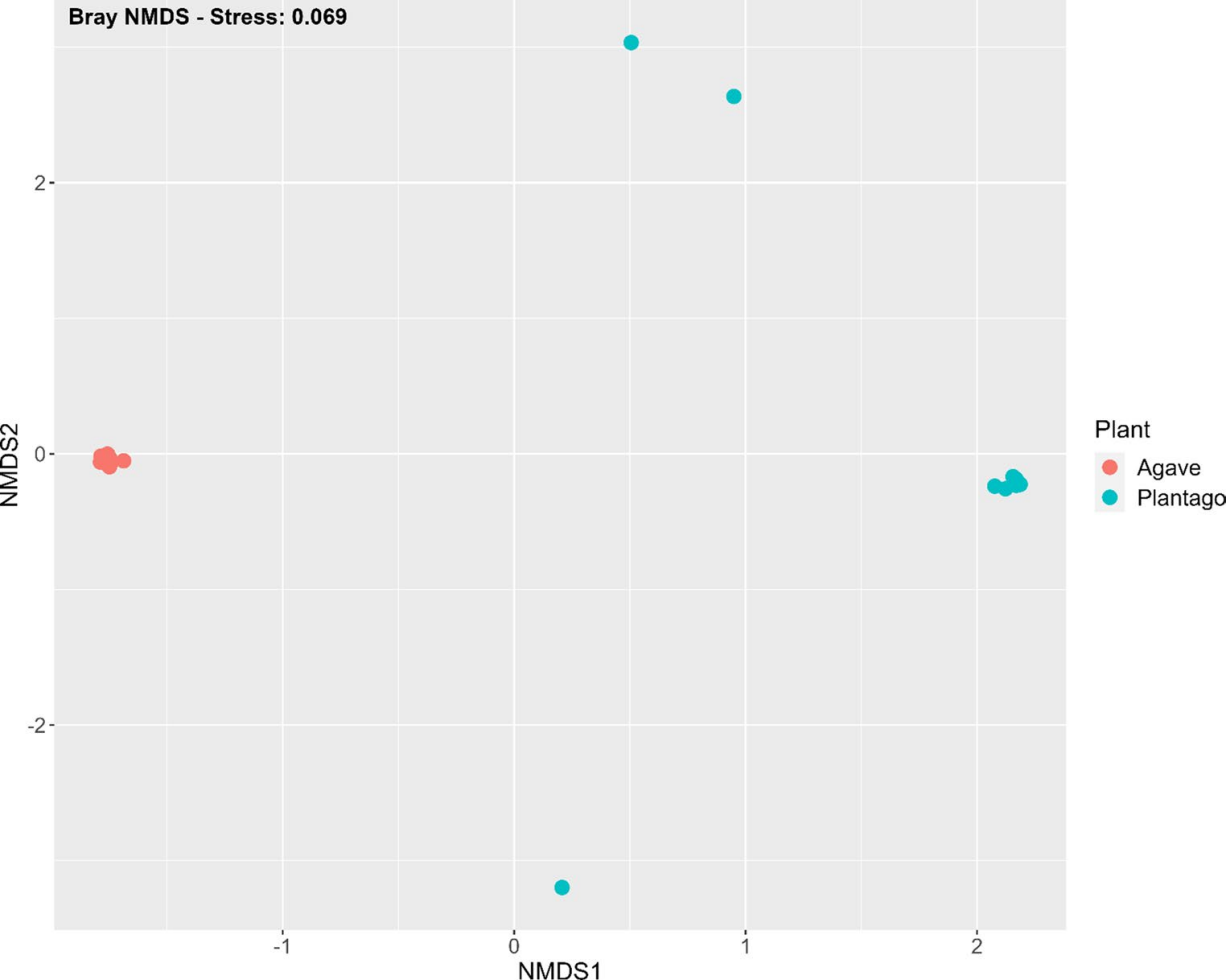
Non-metric multidimensional scaling (NMDS) ordination of arbuscular mycorrhizal fungi community composition in *Agave tequilana* and *Plantago lanceolata *roots, based on Bray-Curtis dissimilarity. Each point represents a sample. The stress value indicates the goodness-of-fit of the ordination, with lower values suggesting a better fit

The non-metric multidimensional scaling (NMDS) plot based on Bray-Curtis dissimilarity illustrates the differences in AM fungal community composition between *A. tequilana* and *P. lanceolata*. This analysis revealed distinct clustering of AM fungal communities associated with each plant species and a low Bray stress value of 0.069. *A. tequilana* samples clustered closely together, showing a high degree of overlap within their group. In contrast, while the majority of *P. lanceolata* samples also formed a tight cluster, three samples were positioned noticeably apart from the main group. These outlying *P. lanceolata* samples were spaced at roughly equal distances from the core cluster (Fig. 3). PERMANOVA analysis supported the observed dissimilarity between the main *A. tequilana* and *P. lanceolata* clusters, confirming that plant species had a significant effect on AM fungal community composition (*p* = 0.0001). In contrast, the effect of location was not significant (*p* = 0.29) (Table 2).

**Table 2:** PERMANOVA results for the beta diversity analysis of AMF community composition associated with *Agave Tequilana* and *Plantago lanceolata*. The analysis was based on Bray-Curtis dissimilarity, with plant species and location as factors and the test used 9999 permutations to determine the significance of the factors

	Df	SumOfSqs	*R*^2^	F	Pr(> F)
Plant	1	3.4837	0.42596	17.011	0.0001
Location	6	1.4180	0.17339	1.154	0.2947
Residual	16	3.2767	0.40065		
Total	23	8.1784	1.00000		

### Taxonomic distribution

**Fig. 4:**
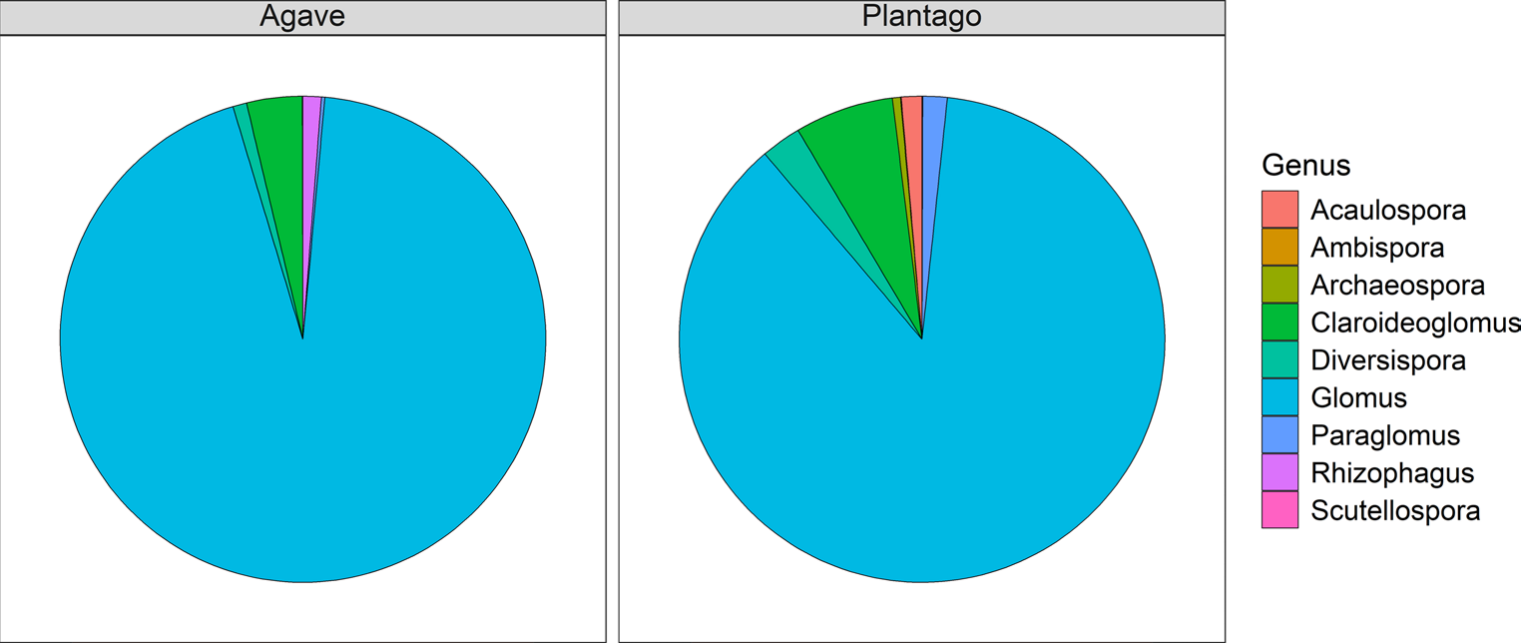
Relative abundance of arbuscular mycorrhizal fungi genera detected in roots of *Agave tequilana* and *Plantago lanceolata*

The genus *Glomus* dominated the AM fungal community structure in both species, accounting for 94% (*A. tequilana*) and 87% (*P. lanceolata*) of the total reads where taxonomy could be assigned (Fig. 4; Table 3). In *A. tequilana*, the second most prevalent genus was *Claroideoglomus* (3.7% of reads, 8 samples), followed by *Rhizophagus* (1.2% of reads, 2 samples) and *Diversispora* (0.9% of reads, 4 samples). *Paraglomus* was found in all 12 samples but made up only 0.2% of all reads. *Ambispora* and *Archaeospora* were minimally represented at 0.01% and 0.03% and *Acaulospora* and *Scutellospora* were completely absent. In contrast, *P. lanceolata* samples exhibited a broader diversity of AM fungal genera. Besides the dominate *Glomus* (87%), all other genera were found in at least two samples with total reads between 0.02 and 6.6%. There was a marked contrast in the presence and abundance of several AM fungal genera between the two host species since *Acaulospora* and *Scutellospora* were detected in *P. lanceolata* but were entirely absent from *A. tequilana*. Similarly, *Claroideoglomus* showed a higher relative abundance in *P. lanceolata*, accounting for 6.6% of reads, compared to just 3.7% in *A. tequilana*. *Diversispora* also followed this trend, at 2.7% for *P. lanceolata* versus only 0.9% in *A. tequilana*. The beta diversity calculated using Bray-Curtis dissimilarity reflected this higher diversity in *P. lanceolata*, with a mean pairwise dissimilarity of 0.60 compared to 0.52 in *A. tequilana* (Table 3).

**Table 3:** Distribution of reads assigned to different genera for *Agave tequilana* and *Plantago lanceolata* samples. The percentage share indicates the proportion of total reads within each plant group and the number of individual samples in which each genus was found. Mean β refers to the mean pairwise Bray-Curtis dissimilarity among samples within each plant group

Plant		Acaulospora	Ambispora	Archaeospora	Claroideoglomus	Diversispora	Glomus	Paraglomus	Rhizophagus	Scutellospora	mean β
Agave	% share	0.0	0.01	0.03	3.7	0.9	93.9	0.2	1.2	0.0	0.5205
n samples		0	3	2	8	4	12	12	2	0	
Plantago	% share	1.4	0.04	0.5	6.6	2.7	87.1	1.6	0.03	0.02	0.6045
n samples		4	3	8	9	10	12	12	2	2	

The top ten ASVs for *A. tequilana* showed ASV2 being most abundant with 52.77% of total reads, followed by ASV4 (5.27%) and ASV6 (4.01%). ASV2 and ASV4 belong to the genus *Glomus* whilst ASV6 has not been assigned taxonomically (Table 4). The majority of the top ten ASVs for *A. tequilana* belong to *Glomus* with only two ASVs belonging to *Claroideoglomus*. For *P. lanceolata*, ASV1 was the most dominant, accounting for 49.86% of total reads, followed by ASV3 (10.95%) and ASV8 (2.63%), all belonging to *Glomus*. Compared to *A. tequilana*, more non-*Glomus* genera are found and in higher abundance, such as ASV13 (*Claroideoglomus lamellosum*, 2.07%) or ASV18 (*Diversispora celata*, 1.20%). ASV 5 and 7, which both belong to *Glomus NES26*, are in the top ten ASVs for both *A. tequilana* and *P. lanceolata*.

**Table 4:** Top 10 amplicon sequence variants (ASVs) associated with *Agave tequilana* and *Plantago lanceolata*, showing total reads of the plant group, percentage of total reads within the plant group, and taxonomic classification down to the species level where available. N/A = no taxonomic classification

Plant group	ASV	Total reads	% of total reads	Family	Genus	Species
Agave	ASV2	594,356	52.77	Glomeraceae	Glomus	SS-G1
	ASV4	59,330	5.27	Glomeraceae	Glomus	Afrothismia
	ASV6	45,134	4.01	*N/A*	*N/A*	*N/A*
	ASV10	31,147	2.77	Glomeraceae	Glomus	*N/A*
	ASV9	31,047	2.76	Glomeraceae	Glomus	NES26
	ASV5	30,694	2.72	Glomeraceae	Glomus	NES26
	ASV7	19,623	1.74	Glomeraceae	Glomus	NES26
	ASV15	18,684	1.66	Claroideoglomeraceae	Claroideoglomus	NES20
	ASV12	18,168	1.61	Claroideoglomeraceae	Claroideoglomus	lamellosum
	ASV17	14,663	1.30	Glomeraceae	Glomus	mosseae
		862,846	76.60%			
Plantago	ASV1	717,130	49.86	Glomeraceae	Glomus	SS-G1
	ASV3	157,502	10.95	Glomeraceae	Glomus	SS-G1
	ASV8	37,806	2.63	Glomeraceae	Glomus	*N/A*
	ASV13	29,787	2.07	Claroideoglomeraceae	Claroideoglomus	lamellosum
	ASV11	28,943	2.01	Claroideoglomeraceae	Claroideoglomus	lamellosum
	ASV5	24,811	1.73	Glomeraceae	Glomus	NES26
	ASV14	24,078	1.67	*N/A*	*N/A*	*N/A*
	ASV7	23,819	1.66	Glomeraceae	Glomus	NES26
	ASV16	20,383	1.42	*N/A*	*N/A*	*N/A*
	ASV18	17,309	1.20	Diversisporaceae	Diversispora	celata
		1,081,568	75.20%			

## Discussion

This study aimed to investigate the AM fungal associations of *A. tequilana* by inoculating potted plants with field-collected soil samples. *P. lanceolata* was used as a trap plant to highlight AM fungal species that were present in the soil but did not colonize *A. tequilana*. Amplicon-based metagenomic sequencing was used to analyze AM fungal colonization and community structure across different soil inoculum sources.

### Experimental design limitations

This study adopted an extensive, landscape‑scale survey design that prioritizes the number of independent inoculum sources (*n* = 12) over within‑source replication. This is a common trade-off in ecological experiments (Hairston 1989). Although statistical power for detecting small treatment effects is reduced, the design captures more diverse AM fungal species and allows first‑order tests of host selectivity across a variety of environmental sites. This is illustrated by the rarefaction curve which shows significant differences in ASV richness per sample, indicating that increasing the number of independently sourced samples captures more overall AM fungal diversity. A clear practical illustration of the value of this experimental design is that, by screening 12 independent inocula, *Acaulospora* was detected in four cases through colonization of *P. lanceolata*, yet it was entirely absent from *A. tequilana* roots. Had the study focused on fewer samples with greater replication, it is likely that inocula containing *Acaulospora c*ould have been missed entirely, obscuring this potential host-specific exclusion. Furthermore, deep sequencing such as in this study (> 50,000 reads per sample) compensates in part for the low physical replication by improving precision of community estimates. Such “breadth-first” experimental designs, which maximize the number of independent samples, have also been shown in published literature to exponentially increase the number of operational taxonomic units (OTUs) detected (Hermans et al. 2019). An alternative approach would have been to combine all field inocula into a single composite sample with increased replication. However, this method risked diluting and potentially excluding less abundant AM fungal species with naturally lower spore densities.

The biggest disadvantage of the experimental design was that fewer replications introduced unmeasured pot-level effects. Despite randomization and consistent greenhouse conditions, this introduces a degree of uncertainty when generalizing findings and doesn’t allow for measuring the effect of AM fungi on agave biomass. However, given that this is the first study to explore AM fungal colonization of *A. tequilana* in its non-native environment, a broader survey was warranted to establish baseline patterns of host-AM fungal associations. This design provides a necessary foundation for more targeted, replicated studies in the future.

### Inoculum soil chemical properties and AM fungal root colonization

The soil collected from around the naturalized agave plants was relatively nutrient poor, with low levels of mineral nitrogen and phosphorus, low EC, and an almost neutral pH. These measurements are the best predictors for successful AM fungal colonization and the values in the collected soil samples are generally favorable for AM fungi (Smith and Read 2008). This natural AM fungal community is likely composed and influenced by symbiotic interactions with both the agave plants and the naturally occurring cover vegetation which are all known to support colonization by AM fungi (Zhu et al. 2000; Becerra et al. 2011; Waddell et al. 2017; Mansfield et al. 2023).

Results showed that agave plants were highly mycorrhizal, with an average of 73% root length colonized across all samples. High colonization rates have been reported in studies on various agave species from Mexico, although they are generally lower at between 20 and 83% root length colonized (Carballar-Hernández et al. 2013; Trinidad-Cruz et al. 2017; Reyes-Jaramillo et al. 2019). This difference could be due to root colonization measured in pot greenhouse studies, such as this one, as opposed to root samples collected in the field. In contrast, *P. lanceolata*, characterized by its rapid growth of hairy roots, showed lower levels of AM fungal colonization which can be interpreted as a dilution effect. It is likely that slow-growing *A. tequilana* roots match the growth speed of AM fungi better than *P. lanceolata* (Smith and Read 2008). The average root length colonized was only 34% between all *P. lanceolata* samples. AM fungal specificity towards host plants was also found in this study using the Bray dissimilarity analysis, with only the plant species being significant (*p* = 0.0001), rather than inoculum location (*p* = 0.2947). This may suggest that the observed differences in AM fungal colonization are more likely driven by the inherent compatibility between the AM fungal community and the plant species rather than environmental conditions across different locations. Plant species as the main driver of AM fungal community structure was reported by Chávez-González et al. (2024) on the CAM plants *A. tequilana*, *A. salmiana* and *Myrtillocactus geometrizans*.

### Alpha and beta diversity

Analysis of the alpha diversity revealed no statistical differences between *A. tequilana* and *P. lanceolata*. However, the beta diversity showed significant differences (*p* = 0.0001), with distinct clusters forming for each plant species. This study used the Shannon alpha diversity, which considers both species richness and evenness within each sample (Henderson 2021). In contrast, beta diversity examines the differences in community composition between samples, accounting for the presence or absence of species as well as the abundance of those species across different samples (Henderson 2021). This explains how there can be differences in beta diversity even when alpha diversity is similar, as beta diversity also captures variations in the AM fungal community composition. The beta diversity analysis provided the first indication that AM fungal communities associated with *A. tequilana* and *P. lanceolata* are very different from each other, which was confirmed by looking at the taxonomic distribution.

### Taxonomic distribution

These differences in the AM fungal community between the two plant species became more pronounced when analyzing the taxonomic range. As hypothesized, *A. tequilana* was predominantly colonized by *Glomus* with 93.9% of all reads belonging to this genus, with a similar high score of 87.1% for *P. lanceolata*. However, *P. lanceolata* showed associations with *Acaulospora* and *Scutellospora* in multiple samples, whereas *A. tequilana* did not. Additionally, *P. lanceolata* had more reads associated with all other genera and a greater diversity overall. This suggests that the *A. tequilana* genotype used in this study, when grown in Australian field soil, associates with a narrower range of AM fungal genera than *P. lanceolata*, pairing predominantly with *Glomus*. In Mexican AM fungal studies, Glomeraceae (*Glomus*) also dominated, based on spore counts from soil collected around agave plants (Carballar-Hernández et al. 2013; Trinidad-Cruz et al. 2017; Reyes-Jaramillo et al. 2019). However, those studies also reported relatively high ratios of Acaulosporaceae (genus *Acaulospora*) and Gigasporaceae (genus *Diversispora*), which were not found here. There was a complete absence of *Acaulospora* in *A. tequilana* roots and very low colonization by *Diversispora*. These differences with the Mexican studies can be explained by functional divergence among AM fungal species across regions. This means that Australian AMF taxa may differ functionally from their Mexican counterparts, making them potentially incompatible with *A. tequilana* (Dodd et al. 1996; Yang et al. 2012).

Another explanation regarding the different distribution of AM fungal families observed between this and the Mexican studies could be attributed to varying AM fungal life strategies and analytical methods. Mexican studies using AM fungal spore extraction identified that up to 35% of them belonged to Acaulosporaceae (Carballar-Hernández et al. 2013) and 27% belonged to Gigasporaceae (Reyes-Jaramillo et al. 2019). However, spore extraction may not accurately represent the actual AM fungal community within the roots. It is known that different AM fungal species have distinct colonization strategies. For instance, *Scutellospora* may rely more on spore production and less on root colonization, resulting in different amounts and proportions of fungal biomass found in roots compared to soil (Hart and Reader 2002; Hart and Klironomos 2002). This could lead to an overrepresentation of these genera in soil spore counts compared to their actual root colonization levels. They also have a larger spore size which might produce certain bias when quantifying AM fungal communities through spore extraction (Brundrett et al. 1996). In contrast, genetic sequencing of root DNA using AM fungal amplicons directly targets the community present and is an objective method of quantification,

When examining the top ten ASVs associated with both plant species, ASV 5 and 7, both assigned to Glomus NES26, were present in *A. tequilana* and *P. lanceolata*, indicating these are generalist species that thrive with different plant hosts. The most common species across both plants was Glomus SS-G1, but within different ASVs, suggesting distinct variants of this species may be adapted to each specific plant. This is not surprising given the well-documented within-species genetic variability and high number of genetically different nuclei within AM fungal spores (Koch et al. 2004). This data also supports the fact that *A. tequilana* predominantly associates with the genus *Glomus* and not with as many genera as *P. lanceolata*. This selective AM fungal relationship could provide insights into *A. tequilana’s* evolutionary advantages in forming symbiotic relationships. A study on leeks found that species from the ancient AM lineages Archaeosporaceae and Paraglomeraceae were less beneficial in stimulating plant growth and nutrient uptake compared to species from the more recently emerged families like Glomeraceae (Säle et al. 2021). *A. tequilana’s* strong preference for the genus *Glomus*, which is phylogenetically more recent, may suggest a specialized adaptation in its physiology and survival strategy under harsh conditions of the southern Americas, for example increasing drought tolerance (Chávez-González et al. 2024). This potential selective relationship, and the symbiotic benefits it brings, warrants further study to determine its functional and agronomic relevance.

### Practical implications

In terms of cultivating agave as an emerging crop in regions outside its native range, the use of commercial AM fungal inoculants for growth promotion may be of limited benefit. These products commonly rely on fast-growing generalist strains such as *Rhizophagus irregularis* (Basiru et al. 2020), which in this study showed little association with *A. tequilana*. Instead, the agave roots were predominantly colonized by *Glomus* species, suggesting limited compatibility with standard inocula. Agave has been promoted as an emerging crop for marginal lands (Corbin et al. 2015; Phillips et al. 2025). These marginal lands might be deprived of a diverse AM fungal community due to low plant biodiversity and adverse growing conditions (Bowles et al. 2017). This means restoring a functional AM fungal community may be critical. In such cases, using locally adapted AM fungal consortia, from well-established *A. tequilana* patches, may be the preferred option. The use of local mixed inoculum has been repeatedly shown to perform better than commercial inoculants (Séry et al. 2016). This is also relevant given agave’s reputation as a drought-resilient crop, which is partly inferred from its symbiosis with AM fungi (Chávez-González et al. 2024). If suitable AM fungi are absent or poorly matched to the host, it is uncertain whether agave would maintain its expected resilience under water-limited conditions.

### Metagenomic method limitations

Metagenomic sequencing was performed on the harvested roots using AM fungal-specific amplicon-based metagenomics. While this method is highly efficient for analyzing AM fungal communities within roots, it has several limitations. Various steps in the process, such as DNA extraction, PCR amplification, and the specificity of the amplicons used, might have favored certain species over others, or even led to the omission of some entirely (Pawluczyk et al. 2015; Ospino et al. 2024). Additionally, metagenomic sequencing can struggle with detecting low-abundant species, which might be underrepresented or missed altogether (Kohout et al. 2014). More bias could also have been introduced through the use of reference database MaarjAM (Öpik et al. 2010) although it remains the most specific AM fungal database to date. To test its suitability for this analysis, we compared taxonomic assignments between MaarjAM and the more general EUKARYOME database (Tedersoo et al. 2024). EUKARYOME could only identify 130 ASVs as Glomeromycota, while MaarjAM classified 1,432 ASVs as Glomeromycota. Notably, all 130 Glomeromycota ASVs identified by EUKARYOME overlapped with MaarjAM classifications, confirming MaarjAM’s higher sensitivity, at least for the AM fungal-specific amplicons used in this study. This is not surprising, given that MaarjAM is a specialist database focused exclusively on AM fungi, with extensive coverage of Glomeromycota lineages and their environmental diversity (Öpik et al. 2010). In contrast, EUKARYOME is a broad, comprehensive reference database that encompasses the full diversity of eukaryotes, including fungi, protists, and metazoans, but likely with less intensive coverage and curation of specific AMF clades (Tedersoo et al. 2024). Additionally, metagenomic sequencing, combined with our specific experimental approach that prioritizes geographic spread over replication, does not permit robust normalization or direct abundance comparisons between *P. lanceolata* and *A. tequilana* based solely on read counts (McMurdie and Holmes 2014). Despite the limitations mentioned in this paragraph, metagenomic sequencing, when interpreted with all limitations in mind, can be a reliable proxy to assess AM fungal community structure (Chagnon and Bainard 2014).

## Conclusion

This study investigated AM fungal associations of *A. tequilana* when grown outside its native range using amplicon metagenomic analysis and *P. lanceolata* as a reference trap plant for each inoculum. Our hypothesis that *Glomus* would predominate in *A. tequilana* root systems in Australian soils was confirmed, mirroring colonization patterns observed in its native Americas. However, unlike in Mexican studies, that found high ratios of Acaulosporaceae, this was not found at all for *A. tequilana*, although it was present in four of the field inocula, as indicated by colonization of *P. lanceolata*. Similarly, Gigasporaceae was found to colonize *A. tequilana* to a lesser degree, as opposed to findings from Mexican studies. These differences could be due to geographical and functional variations of the AM fungal species.

Compared to *P. lanceolata*, *A. tequilana* exhibited a narrower AM fungal association profile. Notably, *R. irregularis*, commonly used in commercial inoculants, was not strongly associated with *A. tequilana*, highlighting important implications for inoculation strategies in commercial cultivation of this economically valuable crop in non-native regions.

## Supplementary Information

Below is the link to the electronic supplementary material.

ESM 1 (DOCX 6.00 MB)

## Data Availability

Data will be made available upon reasonable request.
