# Supplementary material for: Exploring arbuscular mycorrhizal colonization in Agave tequilana: Insights into mycorrhizal partnerships of an emerging crop
Source: Mycorrhiza. 2025 Aug 27;35(5):52. doi: 10.1007/s00572-025-01225-4 (PMC12390875; doi:10.1007/s00572-025-01225-4)
Supplement: Supplementary file 1 — (DOCX 6.00 MB) [file 572_2025_1225_MOESM1_ESM.docx]

**Exploring arbuscular mycorrhizal fungi colonization in *Agave tequilana*: Insights into mycorrhizal partnerships for an emerging crop**

Supplementary information

**Authors:**

^1*^ Salomon M. J., ^1^Burton R. A.

^1^ The Waite Research Institute and The School of Agriculture, Food and Wine, The University of Adelaide, Waite Campus, PMB1 Glen Osmond, SA, 5064, Australia.

Table S1: Dry root and shoot weights of A. tequilana samples from greenhouse experiment and inoculated with collected field soil from various locations across South Australia. Plant grown for 243 days in transparent plastic bags with filter patch to avoid cross contamination with other arbuscular mycorrhizal species.

| **Sample ID** | **Location** | **Root weight dry (g)** | **Shoot weight dry (g)** |
| --- | --- | --- | --- |
| 1 | Glenelg North | 1.5 | 1.7 |
| 2 | Old Newman Nursery | 1.6 | 2.0 |
| 3 | Old Newman Nursery 2 | 1.6 | 1.8 |
| 4 | Windsor | 0.4 | 0.7 |
| 5 | Windsor | 0.9 | 1.1 |
| 6 | Gumeracha | 1.2 | 1.3 |
| 7 | Sellicks Beach | 0.5 | 0.7 |
| 8 | Sellicks Beach | 1.4 | 2.0 |
| 9 | Urrbrae | 1.0 | 1.1 |
| 10 | Urrbrae | 1.8 | 2.3 |
| 11 | Waite | 2.4 | 1.6 |
| 12 | Waite | 1.1 | 1.2 |


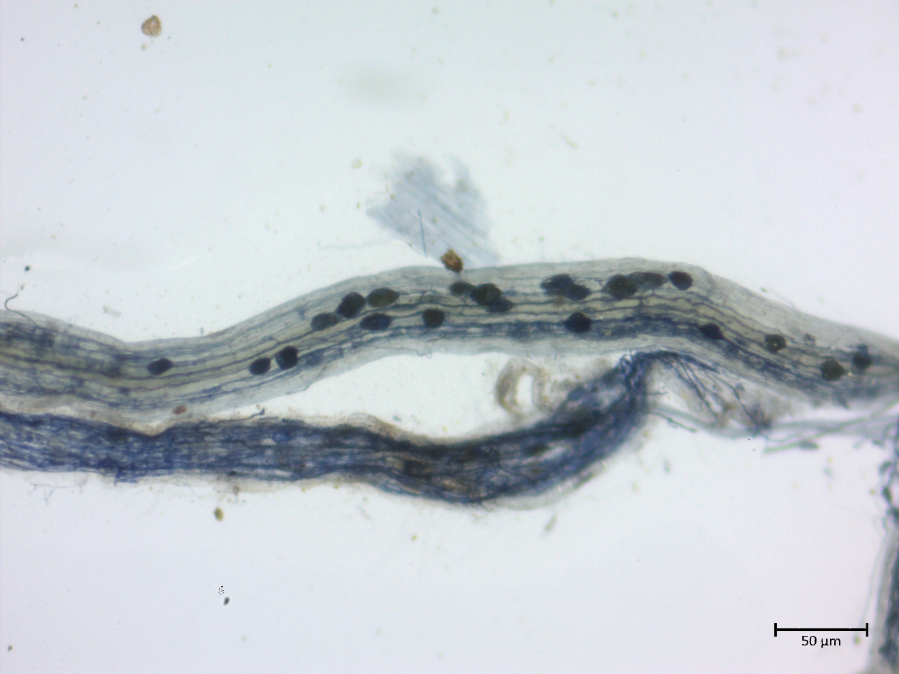


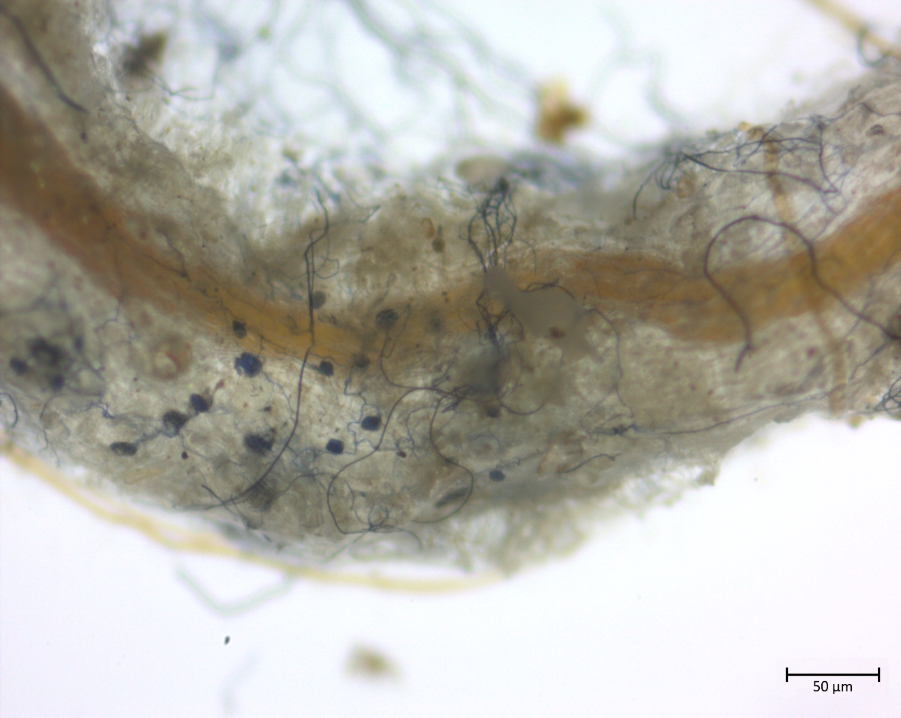


Arbuscule

Hyphae

Intraradical spore

Figure S1: Colonised root piece of A. tequilana (top) and Plantago lanceolata (bottom) depicting arbuscules, hyphae and intraradical spores. Fresh roots collected at harvest and mycorrhizal colonization quantified after root clearing and staining.


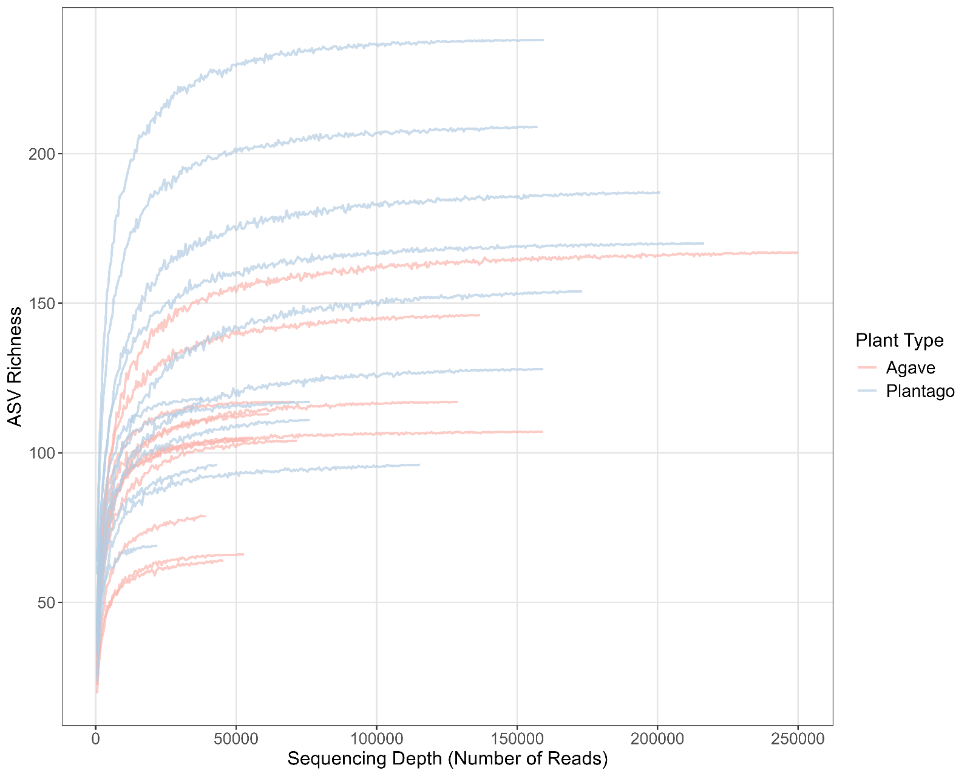


Figure S2: Rarefaction curves showing observed ASV richness as a function of sequencing depth for Agave tequilana and Plantago lanceolata root samples. Each curve represents a single root sample, colours representing plant species.

Table S2: Summary of sequencing depth and ASV richness for Agave tequilana and Plantago lanceolata root samples. Minimum and maximum sequencing depth (reads per sample) and the mean maximum observed ASV richness are shown for each plant species. The minimum depth of 500 reads reflects the threshold set for rarefaction analysis to ensure reliable diversity estimates.

|  | Max depth | Mean max richness |
| --- | --- | --- |
| Agave | 250,076 | 167 |
| Plantago | 216,557 | 170 |
